# Cardiac computed tomography for cardiac masses: a necessity or a luxury?

**DOI:** 10.1177/03000605241306604

**Published:** 2025-02-11

**Authors:** Imane Joudar, Siham Nasri, Imane Kamaoui, Imane Skiker

**Affiliations:** 1 Faculty of Medicine and Pharmacy, Mohammed First University, Oujda, Morocco; 2 Department of Radiology, Mohammed VI University Hospital Mohammed I University, Oujda, Morocco; 3 Mohammed First University, Faculty of Medicine and Pharmacy, LAMCESM, Oujda, Morocco

**Keywords:** Cardiac mass, computed tomography, contrast enhancement, benign tumor, malignancy, non-neoplastic mass

## Abstract

Cardiac masses present a considerable diagnostic challenge because of their diverse causes and potential clinical implications. Traditional imaging methods, such as transthoracic echocardiography and transesophageal echocardiography, are crucial for initial assessments owing to their accessibility, but they have a major limitation represented by inter-operator variability. Therefore, cardiac computed tomography (CT) has become an indispensable adjunct, providing detailed anatomical information and tissue characterization. This review examines the prevalence, categorization and diagnostic benefits of cardiac CT in the evaluation of cardiac masses. The high-resolution imaging and multiplanar features of cardiac CT allow in-depth assessment of the structure, location and enhancement patterns of masses, helping to distinguish benign from malignant masses and guide clinical decision-making. Specific imaging features of benign and malignant tumors, as well as non-neoplastic masses, are discussed, highlighting the role of CT in overall cardiac assessment. This article highlights the importance of CT in surgical preparation, risk assessment and ongoing monitoring, and highlights its effect on improving patients’ outcomes. With continued advances in CT technology, the integration of this modality into routine clinical practice should improve the accuracy of diagnosis and management of cardiac masses, strengthening the essential role of cardiac CT as a vital component of contemporary cardiovascular imaging.

## Introduction

Cardiac masses represent a diagnostic challenge because of their diverse origins and clinical implications. Traditionally, transthoracic echocardiography and transesophageal echocardiography have been fundamental tools for the initial assessment of these masses because of their accessibility, real-time capabilities and absence of ionizing radiation. Nevertheless, computed tomography (CT) has gradually emerged as an essential complementary modality, providing unrivalled advantages in terms of precise anatomical assessment and tissue characterization. The superiority of CT results from its ability to provide high-resolution images of the complete heart and surrounding structures, regardless of the patient’s anatomy or acoustic window limitations encountered during echocardiography.^1^ This capability is particularly crucial for defining complex cardiac masses located in difficult anatomical sites, such as the pericardium or close to major vessels. In addition, CT excels in tissue characterization because of its ability to analyze tissue density, enhancement type and perfusion characteristics. These attributes are essential in distinguishing between benign and malignant masses, detecting thrombi and assessing infectious or inflammatory processes that may resemble neoplastic tissue growth.^2^

In addition to improving the diagnostic accuracy, cardiac CT greatly affects clinical decision-making and patient care. CT facilitates preoperative preparation by providing precise anatomical maps and three-dimensional reconstructions that guide interventions, such as biopsies, tumor excisions or device insertions. In addition, CT plays a major role in risk assessment, particularly the risk of embolization or hemodynamic compromise involved in cardiac masses.^3,4^ This role includes not only detailed anatomical information, but also the ability to identify associated complications, such as pericardial effusion, vascular invasion and infiltration into adjacent cardiac structures.^5^ The full information provided by CT considerably contributes to the multidisciplinary approach required for managing complex cardiac masses, ensuring more informed clinical decisions and improving patients’ outcomes. One of the main limitations of cardiac CT is the high rate of irradiation, especially in pediatric patients, and the risk of renal failure and allergy following use of the iodine contrast medium. Therefore, other imaging modalities such as cardiac MRI in specific patient populations should be considered.

This narrative comprehensive review provides an in-depth examination of the importance of cardiac CT in the evaluation of cardiac masses, and highlights its diagnostic efficacy, clinical significance and comparative advantages over conventional imaging techniques. This review aims to enrich clinicians’ and researchers’ understanding of the rapidly evolving field of cardiac imaging through a detailed description of the imaging characteristics of various benign, malignant and non-neoplastic cardiac masses.

This review was guided by the Scale for the Assessment of Narrative Review Articles (SANRA).^6^

## Cardiac CT scanning protocols

The cardiac CT protocol is adapted to the location and type of the suspected mass and to the orientation of clinical findings. The first step is to ensure electrocardiographic gating, the aim of which is to reduce cardiac movements, which are a source of artefacts. Regarding triggering, prospective triggering is preferable because of the lower exposure to radiation. However, when assessment of the mobility of the mass is envisaged, as well as valve dynamics using cine images, retrospective triggering is recommended.^7^

Beta-blockers and nitroglycerine are not necessary for evaluating cardiac masses. The size of the patient determines the current and the tube voltage. To perform an enhancement study, a triphasic injection protocol is preferred to visualize all of the cardiac structures and minimize artefacts. This protocol consists of an initial injection of contrast at a rate of 5 to 7 mL/s to analyze the cavities of the left heart, followed by an injection at a slower rate of 2 to 4 mL/s to analyze the structures of the right heart.^8^ This phase can be carried out at the same flow rate as the first phase, but it should be mixed with physiological saline. Finally, the last step aims to minimize streak artifacts by injecting a bolus of saline. The images are reconstructed to a submillimeter thickness (0.5–0.75 mm), with a small field of view (200–250 mm) for the heart.^9^

## CT cardiac approach and differential diagnosis

The evaluation process begins with the patient’s history and clinical context, which are essential in determining the appropriate imaging approach. Depending on the suspected condition and clinical demands, different CT protocols may be used, such as non-contrast, contrast-enhanced and gated protocols aimed at assessing cardiac function and structure.^10^

**Figure 1.**
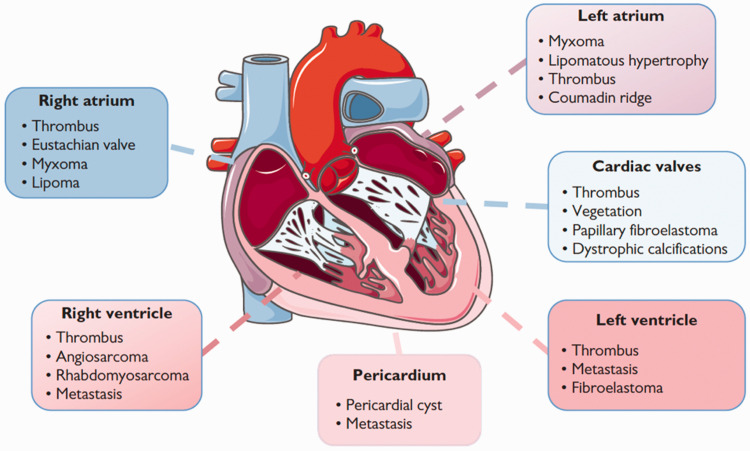
Distribution of the subtypes of cardiac masses according to their localization (used with permission).

**Figure 2.**
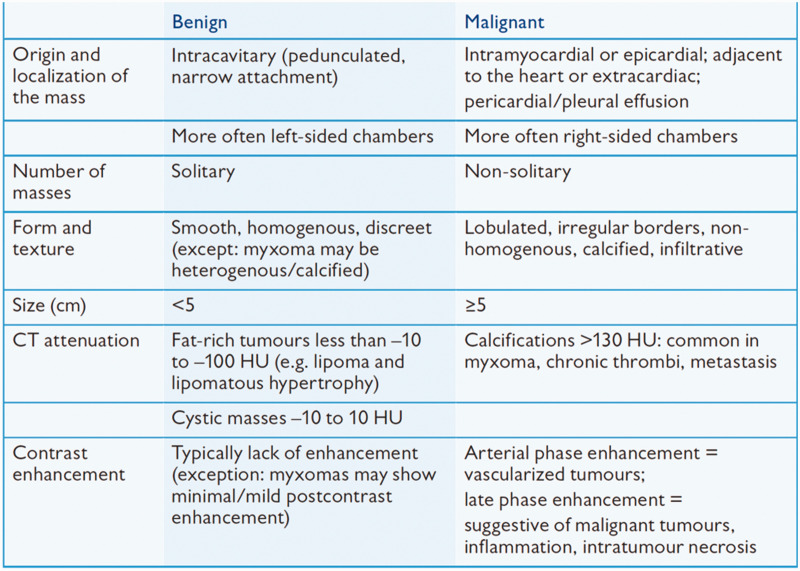
CT characteristics of benign versus malignant cardiac masses (used with permission). CT, computed tomography; HU, Hounsfield units.

From a morphological point of view, cardiac CT provides detailed information on the dimensions, configuration and limits of the mass, enabling the differentiation of benign tumors (e.g., myxoma and fibroma) from malignant tumors (e.g., cardiac sarcoma and metastases). In contrast, malignant masses often appear invasive, with irregular margins and varied enhancement patterns on contrast-enhanced CT scans. Precise localization of the mass within the heart is another essential aspect of cardiac CT. This precise determination of the anatomical site, whether in the atria, ventricles or pericardium, is essential for a differential diagnosis and informs decisions regarding subsequent interventions, such as surgery and biopsy (Figure 1). The assessment of enhancement patterns on contrast-enhanced CT scans provides additional diagnostic information.^11^ Benign masses such as myxomas typically show prominent enhancement, while thrombi may show peripheral enhancement because of their vascularity. However, malignant masses typically show irregular and diverse enhancement patterns, reflecting their aggressive nature and variable blood supply (Figure 2).^12^

In addition, cardiac CT allows assessment of the relationship of the mass to adjacent structures, such as the coronary arteries, heart valves and myocardium. This assessment helps determine whether the mass is causing compression, invasion or displacement of adjacent tissues, which influences therapeutic decisions and surgical planning.

## Epidemiology and classification

Cardiac masses, although uncommon, pose considerable clinical problems because of their potential effect on cardiac function Autopsy studies have suggested that the prevalence of cardiac masses is between 0.002% and 0.33%, which indicates their rarity. These masses can be classified into benign tumors, malignant tumors and non-neoplastic masses, such as thrombi and vegetations.^13^

**Figure 3.**
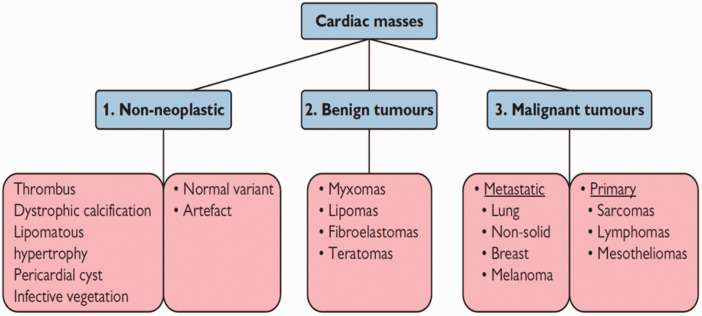
Classification of cardiac masses according to World Health Organization classification (used with permission).

In 2021, the World Health Organization revised the classification of cardiac masses, encompassing benign tumors, tumor-like lesions, malignant tumors and pericardial tumors (Figure 3). The classification of cardiac tumors is based on the distinction between primary and secondary forms, which is a fundamental distinction for clinical and diagnostic applications.^14^ The prevalence of primary cardiac tumors is estimated to be 1/2000 autopsies, while secondary tumors have a prevalence of 1/100 autopsies, resulting in a secondary/primary ratio of 20:1. This ratio highlights the relatively higher incidence of secondary cardiac tumors in autopsy findings. Of all primary cardiac tumors, approximately 10% are malignant, while the majority (90%) are benign in nature, and myxomas are the most common benign tumors, particularly in adults.^15^ Benign tumors such as myxomas are mainly found in middle-aged adults, with a slight female predominance, while rhabdomyomas are typically diagnosed in infants and young children. Malignant tumors such as angiosarcomas tend to occur in individuals between the third and fifth decades of life, with no marked sex preference. Secondary cardiac tumors match the epidemiology of their primary cancers, with a higher occurrence in adults because of the prevalence of metastatic cancers in this age group.^16^

Myxomas account for approximately 50% of all benign cardiac tumors in the adult population, with a lower occurrence in children.^12^ In particular, rhabdomyoma is the predominant benign cardiac tumor in children, accounting for 40% to 60% of cases in this population. In addition, various other benign cardiac tumors, such as fibromas, lipomas, hemangiomas, papillary fibroelastomas, cystic tumors of the atrioventricular node and paragangliomas, have been documented, contributing to the diverse spectrum of cardiac masses. The remaining subset of primary cardiac tumors, ranging from 10% to 20%, are classified as malignant and are usually identified as sarcomas on a pathological examination.^17^

Regarding cardiac sarcomas, angiosarcomas and unclassified sarcomas are the most prevalent, collectively accounting for 76% of all cardiac sarcomas, and angiosarcomas are the predominant subtype. Rhabdomyosarcoma occurs predominantly in children. In addition, leiomyosarcoma, synovial sarcoma, osteosarcoma, fibrosarcoma, myxoid sarcoma, liposarcoma, mesenchymal sarcoma, neurofibrosarcoma and malignant fibrous histiocytoma are among the various cardiac sarcomas identified in clinical practice.^18^

Secondary cardiac tumors, originating from other primary sites, exceed primary cardiac tumors in frequency. The most common primary sites are the lungs, breast, kidney and skin, with tumors usually spreading to the pericardium, myocardium or endocardium. Non-neoplastic masses in the heart, such as thrombi and vegetations, are also of clinical importance. Thrombi can develop in the heart chambers or on the heart valves, and are often associated with conditions, such as atrial fibrillation, myocardial infarction and heart failure. Vegetations are masses composed of platelets, fibrin, micro-organisms and inflammatory cells, and are commonly observed in infective endocarditis.^19,20^

**Figure 4.**
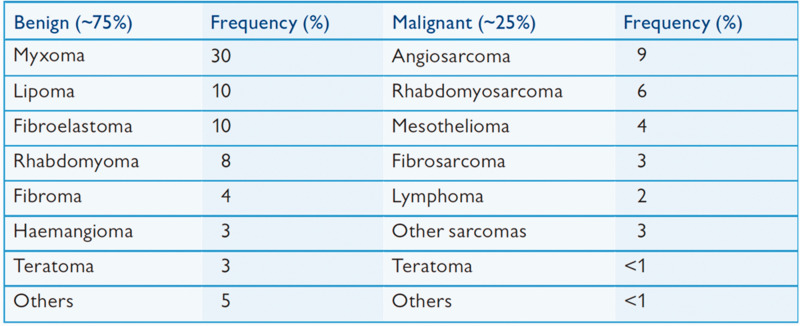
Frequency of the principal primary cardiac masses (used with permission).

Figure 4 shows the frequency of the principal primary cardiac masses.

## Benign cardiac tumors

Benign cardiac tumors, although uncommon, comprise a range of classifications, such as myxomas, fibromas, lipomas and papillary fibroelastomas, each with distinct characteristics identifiable through cardiac CT imaging. This detailed imaging plays a crucial role in the differential diagnosis and subsequent treatment strategies because of the unique attributes of each tumor.

### Myxomas

**Figure 5.**
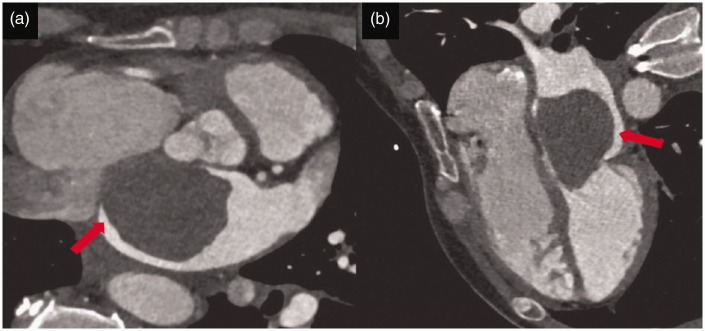
(a) Axial and (b) sagittal cardiac computed tomography images showing a large left atrial myxoma (red arrows) with diastolic prolapse into the left ventricle (used with permission).

Myxomas are the most common form of benign cardiac tumor and are usually located in the left atrium, from the interatrial septum onwards. When visualized by cardiac CT, myxomas usually present as solitary masses with heterogeneous enhancement patterns and low attenuation values. This heterogeneity of enhancement is the result of the tumor’s composition, which incorporates elements of hemorrhage, necrosis and calcification. Myxomas are often intracavitary, pedicle-like, connected to the septum by a stalk and may show a “ball valve” mechanism, causing the tumor to move to the mitral valve and obstruct it during diastole. This situation sometimes results in symptoms of mitral valve obstruction (Figure 5). These symptoms include syncope, dyspnea and, in the most severe cases, sudden cardiac death. The mobility of myxomas can also cause embolisms, when fragments become dislodged and migrate to other areas of the body, potentially causing strokes or other embolic complications.^21^

### Fibromas

**Figure 6.**
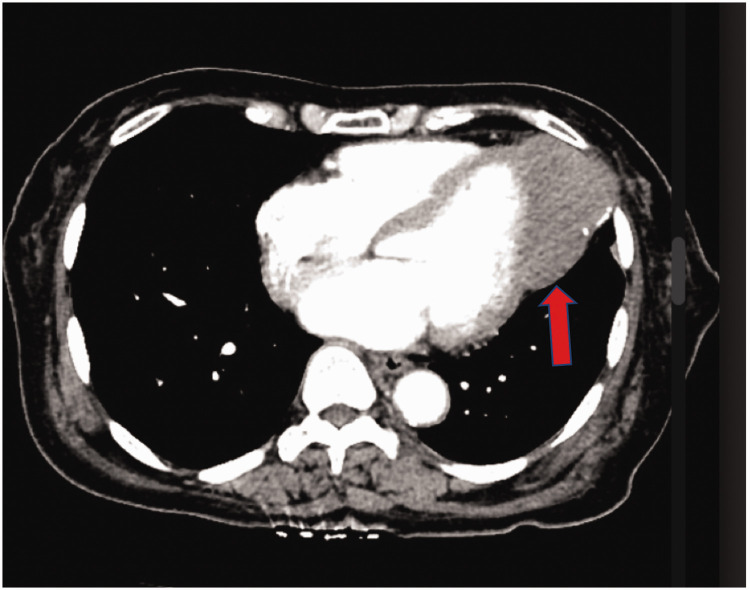
Axial cardiac computed tomography image showing large isodensity in left myocardial masses with calcifications, which suggests cardiac fibroma (red arrow) (used with permission).

Fibromas are another benign cardiac tumor variant, and they are predominantly located in the ventricular myocardium, particularly in pediatric cases. When viewed on cardiac CT scans, fibromas appear as solid, uniform masses, and are homogeneous masses of soft tissue that are generally well defined, with notable contrast enhancement owing to their fibrous tissue density. Unlike myxomas, fibromas are not pedunculated but are rather embedded within the myocardial wall (Figure 6).^22^ The inflexible and non-compressible nature of fibromas can lead to arrhythmias and conduction disorders if they encroach upon the cardiac conduction system. While generally devoid of symptoms, large fibromas can elicit symptoms, such as heart failure or outflow tract obstruction, associated with their dimensions and placement.

### Lipomas

**Figure 7.**
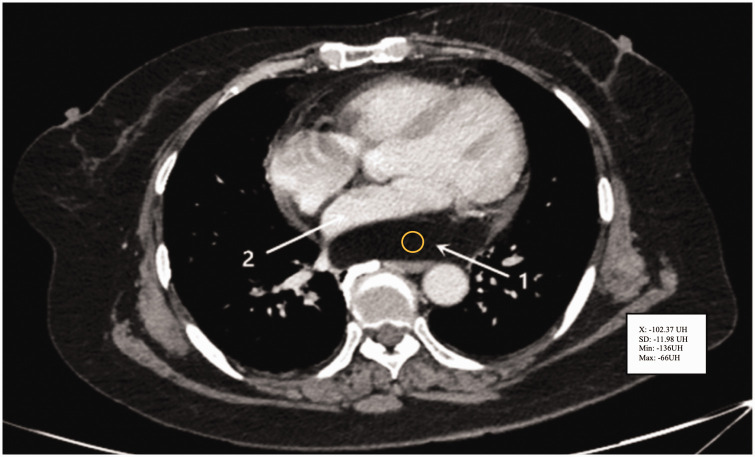
Axial cardiac computed tomography image showing a large low-density mass located on the posterior wall (1) of the left atrium (2) without enhancement, which suggests cardiac lipoma (used with permission).

Lipomas consist of mature adipose tissue and can emerge in various regions of the heart, encompassing the epicardial, endocardial or myocardial domains. When lipomas are visualized on cardiac CT scans, they are identified by their well-defined, isolated, intra-cavitary nature with low attenuation attributed to their adipose content, and without contrast enhancement (Figure 7).^16^ Lipomas typically remain asymptomatic and are frequently incidentally discovered during imaging for unrelated reasons. Nevertheless, substantial lipomas can induce symptoms by compressing adjacent cardiac structures, leading to chest discomfort, arrhythmias or even heart failure.

### Papillary fibroelastomas

**Figure 8.**
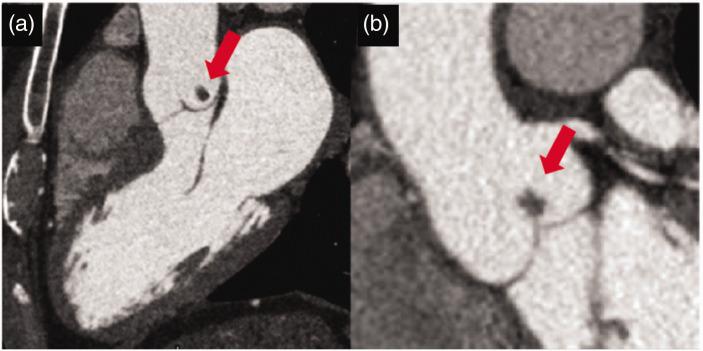
Cardiac computed tomography images showing a small homogeneous non-contrast-enhanced mass on the aortic side of the left coronary cusp in association with a papillary fibroelastoma (red arrows). (a) Long-axis view of the three chambers and (b) Long-axis view of the aorta (used with permission).

Papillary fibroelastomas are avascular tumors typically located on cardiac valves, notably the aortic and mitral valves. Papillary fibroelastomas present as small, mobile masses with tentacle-like extensions, with low density and non-contrast enhancement on cardiac CT scans (Figure 8).^23^ The mobility and positioning of papillary fibroelastomas make them susceptible to embolization, which can lead to transient ischemic attacks or strokes. These tumors generally remain asymptomatic until they cause embolic events or valve dysfunction.

## Malignant cardiac tumors

Malignant cardiac tumors, such as sarcomas and metastases, present challenging diagnostic issues that require careful characterization to make well-informed decisions about management strategies.

### Sarcomas

**Figure 9.**
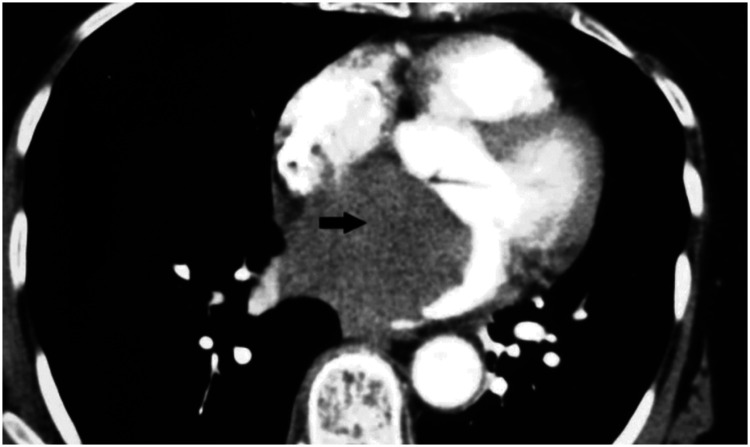
Cardiac computed tomography image shows a large, homogeneous, non-contrast-enhanced mass with low attenuation at the right atrium, suggesting cardiac angiosarcoma (black arrow) (used with permission).

Cardiac sarcomas, although uncommon, are aggressive primary cancers that require thorough evaluation to determine the full extent of their presence and effect on the heart. When viewed on cardiac CT, sarcomas typically present as invasive masses with irregular, unclearly defined borders, low focal attenuation and heterogeneous contrast enhancement consistent with their histological diversity and aggressive behavior.^24^ Cardiac sarcomas frequently extend into several cardiac chambers and may extend into adjacent structures, such as the pericardium and major vessels. These features indicate the infiltrative potential of sarcomas and the requirement for thorough imaging to accurately delineate the full extent of this disease (Figure 9).

### Primary cardiac lymphoma

On a cardiac CT scan, cardiac lymphomas are generally localized in the right ventricle and present as hypo- or isoattenuating masses with heterogeneous enhancement and no particular feature on post-contrast images. Calcification is extremely rare in primary cardiac lymphomas and its presence suggests the possibility of other diagnoses or that radiotherapy should be performed.^25^

The infiltrative nature of primary cardiac lymphomas is well known. They extend along the epicardial surface and tend to involve the coronary arteries and the aortic root. Primary cardiac lymphomas do not usually involve the valves, although rare cases have been described.^12^ Pericardial thickening and pericardial effusion are observed in primary cardiac lymphomas. The amount of pericardial effusion can be large and may even lead to tamponade.^26^

### Metastatic tumors

**Figure 10.**
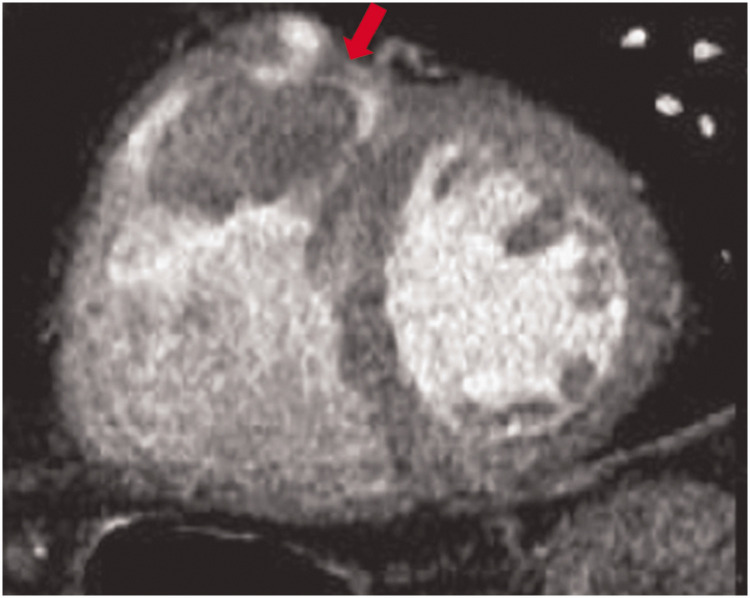
Cardiac computed tomography short-axis image shows a lobulated mass (red arrow) attached to the right ventricular outflow-tract in a patient with known hepatocellular carcinoma (used with permission).

Cardiac metastases are secondary tumors and usually manifest as multiple nodular lesions in the heart. The imaging characteristics of cardiac metastases are affected by the type of primary tumor, and the lungs, breast and skin are common sites. Intramyocardial metastases generally spread hematogenously, while pericardial metastases extend lymphatically. On cardiac CT, metastases often present as an intramyocardial and rarely intracavitary mass, with hypoattenuation and variable heterogeneous contrast enhancement due to disparities in blood supply and tissue composition in relation to the surrounding myocardium (Figure 10). The presence of multiple lesions distributed in different cardiac cavities provides crucial evidence of the underlying primary cancer, guiding further investigations and therapeutic decisions. Imaging plays an essential role in identifying the site of the primary tumor and accurately assessing the degree of cardiac involvement because of the diverse origins of cardiac metastases.^27^

## Non-neoplastic tumors

Non-neoplastic cardiac tumors, also known as pseudo-tumors, include a wide range of benign masses, such as thrombi, pericardial cysts, dystrophic calcification of the mitral annulus and vegetations. Despite the benign nature of non-neoplastic cardiac tumors, these lesions can have considerable clinical implications because of the possibility of obstruction or embolization. The use of cardiac CT is crucial for careful evaluation and differentiation of these masses within the cardiac structure.

### Thrombi

**Figure 11.**
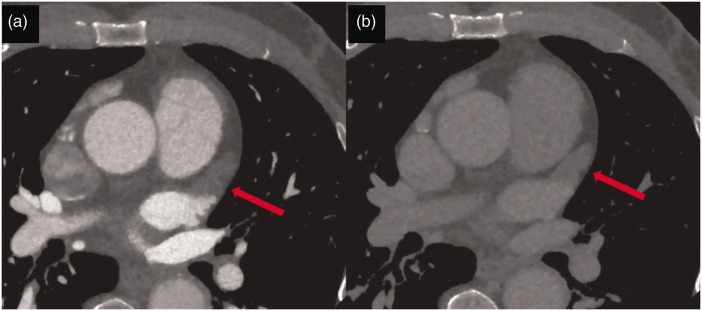
(a) Axial cardiac computed tomography image shows the presence of thrombus in the left atrial appendage (red arrow) and (b) A late scan showing persistence of low attenuation at a repeat scan 60 s after the first scan (used with permission).

Cardiac thrombi are blood clots that usually develop in the heart chambers and are frequently observed in the left atrium, particularly in patients with atrial fibrillation or mitral valve disease. When thrombi are visualized by a cardiac CT scan, they appear as hypoattenuating masses in the heart chambers and are similar in density to blood, but without contrast enhancement. These thrombi are generally located in the left atrial appendage or along the atrial walls (Figure 11). The absence of contrast enhancement is an essential distinguishing feature of thrombi, facilitating their differentiation from other intracardiac masses.^28^

### Dystrophic calcification of the mitral annulus

**Figure 12.**
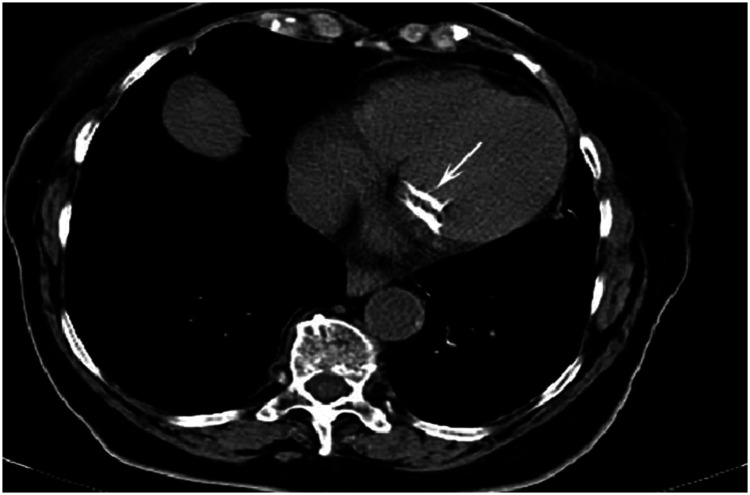
Axial cardiac computed tomography image shows the presence of annular mitral calcification (white arrow) (used with permission).

Typically, dystrophic calcification of the mitral annulus involves calcareous deposits on myocardial tissue, mainly around the mitral annulus.^28^ Cardiac CT enables precise assessment of the extent of these calcifications (focal or diffuse), and diffuse calcifications appear spontaneously hyperdense, and sometimes have central necrosis with hypoattenuation without contrast enhancement (Figure 12).

### Vegetation

Cardiac CT is important for evaluating and diagnosing valvular vegetations, with a sensitivity of 97% and specificity of 88% compared with transesophageal echocardiography. On CT, vegetation is defined as a hypodense, circular, irregular or longitudinal mass attached to the valve leaflets or endocardium (“mural vegetation”). When these vegetations are chronic, they can become calcified.^29^

## Future recommendations

Dual-energy CT represents one of the technical advances that has enabled advanced characterization of tissues and perfusion. An example of this characterization is that this technique can distinguish myxomas from thrombi on the basis of iodine concentrations, despite the fact that native Hounsfield units between the thrombus and myxoma are the same.^30^

Additionally, three-dimensional reconstruction from high-resolution two-dimensional images is extremely useful for pre-surgical planning because it helps surgeons to acquire another level of understanding by simply holding, rotating and manipulating a simulated model of the anatomy to help plan an operation before it starts. This information can lead to a higher level of confidence starting surgery, a shorter time in the operating theater and less blood loss.^7^

Finally, photon-counting CT represents a promising technological advance that uses photon-counting detectors. This technique provides several advantages, such as better signal and contrast behavior, better spatial resolution, reduced exposure to radiation and reduced electronic noise, compared with traditional CT scanners. These advantages enable more detailed qualitative assessment of coronary plaques, better quantification of coronary calcium, and individualization of cardiac masses by determining the extracellular volume of the myocardium, and more detailed characterization of the myocardium and pericardial adipose tissue.^31^

## Conclusion

Cardiac CT has become an essential tool for thorough evaluation of cardiac masses, providing complex anatomical visualization and precise tissue characterization. Despite the essential role of echocardiography in the initial assessment, complementary application of cardiac CT helps to improve diagnostic accuracy and affect clinical management decisions. Ongoing advances in CT technology, such as improved spatial resolution and reduced radiation exposure, offer the potential to further improve the examination of cardiac masses.

## Data Availability

The data underlying this article are available in the article.
